# Natural exosome-like nanoparticles derived from ancient medicinal insect *Periplaneta americana* L. as a novel diabetic wound healing accelerator

**DOI:** 10.1186/s12951-023-01923-1

**Published:** 2023-05-26

**Authors:** Qian Liao, Lijun Su, Lan Pang, Jiaxin Li, Hui Li, Jingjing Li, Yuling Liu, Jinming Zhang

**Affiliations:** 1 State Key Laboratory of Southwestern Chinese Medicine Resources, Pharmacy School, Chengdu University of Traditional Chinese Medicine, No.1166 Liutai Avenue,Wenjiang District, Chengdu, 611137 China; 2 Institute of Chinese Materia Medica, China Academy of Chinese Medical Sciences, Beijing, 100700 China; 3 Department of Rehabilitation Sciences, Faculty of Health and Social Sciences, Hong Kong Polytechnic University, Hong Kong SAR, China

**Keywords:** *Periplaneta americana* (L.), Exosome-like nanoparticles, Proteomics, Small RNA, Wound healing, Autophagy

## Abstract

Along with the recognized therapeutic outcomes of regenerative medicine, extracellular vesicles and their exosome subsets have become an alternative option for wound healing. *Periplaneta americana* L. (PA), an ancient and traditional medicinal insect, has been around for 300 million years, and displays magic formidable vitality and environmental adaptive ability. The linkage between intrinsic amputation regeneration feature and the acknowledged wound healing medicinal benefit of PA has never been revealed. Herein, inspired by the ability of exosomes to participate in the interkingdom communication, we explored whether this effect was ascribed to PA derived exosome-like nanoparticles (PA-ELNs). PA-ELNs were extracted by differential velocity centrifugation approach and characterized by DLS, NTA and TEM. Their cargoes were analyzed by LC-MS/MS proteomics and small RNA-seq analysis. The wound healing activity was verified in vivo and in vitro. PA-ELNs with a concentration of 2.33x10^9^±6.35x10^7^ particles/mL exhibited a lipid bilayer-bound membrane structure with an average size of 104.7 nm. Furthermore, the miRNA cargoes in PA-ELNs participate in some wound healing related signal pathways such as TGF-beta, mTOR, and autophagy. As expected, the in vitro tests indicated that PA-ELNs were apt to be internalized in HUVECs, L929 and RAW 264.7 cells and contributed to cell proliferation and migration. Most importantly, we demonstrated that the topical administration of PA-ELNs could remarkably accelerate wound healing in a diabetic mouse model, and was involved in anti-inflammatory, re-epithelialization and autophagy regulation. This study provides clear evidence for the first time that PA-ELNs, as diabetic wound healing accelerators, are the “bioactive code” of this ancient medicinal insect.

**Graphical abstract:**

## Highlights

*Periplaneta americana* L. derived exosome-like nanoparticles (PA-ELNs) as a novel wound healing accelerator was isolated and proved.

Protein and small RNA components in PA-ELNs were characterized by omics technology.

PA-ELNs remarkably accelerated diabetic wound healing, and were involved in anti-inflammatory, re-epithelialization and autophagy regulation in this process.

## Graphical abstract

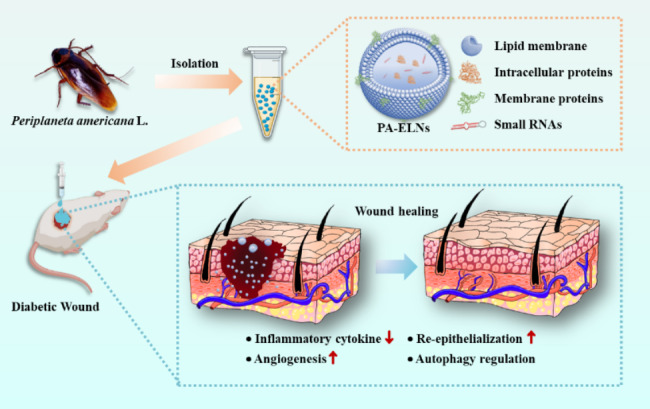

## Introduction

Diabetes mellitus wound, among the most frequent diabetic complications, is a complex metabolic disorder that leads to expensive medical expenses and poor quality of life for millions of people around the world. Latterly, mesenchymal stem cells (MSCs) have exhibited remarkable therapeutic potential for various kinds of wounds, and are involved in direct differentiating into skin cells, immunoregulation, paracrine effects to promote vascularization, and so on [1–3]. However, emerging evidences on the potential risks caused by stem cell therapy, such as severe complications and immunological rejection, vastly impede the clinical translational applications. Interestingly, exosomes, as secreted extracellular vesicles with nanoscale phospholipid bilayer structure, have been reported to facilitate cell-cell communication and signal transduction by transferring their cargoes including RNA and protein to recipient cells [4]. Exosomes derived from MSCs have been shown to facilitate wound healing by contributing to cell migration, proliferation, angiogenesis, collagen deposition, re-epithelialization processes and regulating macrophage polarization [5–7]. Nevertheless, the application of MSCs-Exo is also greatly hindered because of the high cost. To develop the safe, easily-prepared, and cost-effective therapeutic agents for chronic wound healing has become a global challenge.

The application of natural products or herbal medicines, which act in multiple wound healing mechanisms including anti-inflammatory, angiogenesis stimulation, fibroblast proliferation, extracellular matrix formation, immunomodulatory, etc., has received considerable researchers’ concern over the years [8]. Apart from these recognized medicinal plants, insects and their extracts have also been used in folk medicine for a long time [9]. Some bioactive components isolated from insect body, excretions and exudations have also been acknowledged as effective wound healing accelerators, such as propolis, chitosan and sericin. American cockroach (*Periplaneta americana* L., PA) is an ancient and traditional medicinal insect, that may have existed on the earth approximately 300 million years ago and was recorded in many TCM classics written two thousand years ago. Many pharmacological effects of PA have been demonstrated [10–12]. Particularly, the wound healing benefits of PA on skin wounds have been utilized both in ethnic use and in clinics, such as a commercial Chinese patent medicine “Kangfuxin Liquid” comprised of PA extracts [13, 14]. The mechanisms of PA and its extracts in wound healing are involved in the whole physiological process, including promoting kerationcytes/fibroblasts proliferation and migration, stimulating EGF and VEGF secretion, and accelerating wound closure, collagen synthesis and angiogenesis [12, 15]. In previous studies, the amino acids, polysaccharides, and glycoproteins in PA have been deemed to be relevant to wound healing benefits [16–18]. However, the effective material basis in PA is still unknown.

Excitingly, exosome-like nanoparticles (ELNs) derived from animal and plant products were found to exhibit various bioactivities in immunological modulation, antitumor activity, tissue regeneration, and anti-inflammation [19–21]. Some nature-derived ELNs have been isolated from milk, honey, grape, ginger, garlic, turmeric, and other natural products, in which protein, lipid, RNA and other substances are rich. As one of the most ancient insects in the world, which may exist on the earth approximately 300 million years ago, PA possesses strong environmental adaptation and developmental plasticity. Most surprisingly, PA has a strong capability of limb regeneration with contributions from wound healing signaling pathways such as Decapentaplegic, Jun N-terminal kinase, Grainy head, Wingless, Notch, and so on in the PA genome [22]. Besides, whether the intrinsic tissue regeneration code of the PA genome is relevant to its wound healing benefits is interesting and attractive. Therefore, in this study, we isolated ELNs from PA for the first time, in which these contained protein and RNA cargoes were decoded. As expected, PA-ELNs promoted wound-associated cells proliferation and migration in vitro and accelerated diabetic mice wound healing, which was involved in inflammation, re-epithelialization and autophagy regulation. This study sheds light on the application of animal resource-derived ELNs in wounds therapy.

## Materials and methods

### Materials

Dried nymphs of PA were obtained from Sichuan Good Doctor Pharmaceutical Group Co., Ltd. Coomassie brilliant blue fast staining solution was obtained from Beyotime Technology Co., Ltd (Shanghai, China). DL-Dithiothreitol and iodoacetamide were obtained from Adamas Pharmaceuticals, Inc. RIPA lysis solution, BCA protein concentration determination kit, 5x reduced protein loading buffer, sodium dodecyl sulfate‒polyacrylamide gel electrophoresis (SDS‒PAGE) gel preparation kit and protein marker were all obtained from Servicebio Technology Co., Ltd (Wuhan, China). Phosphate buffer (powder, pH 7.2–7.4) was acquired from Livning Biotechnology Co., Ltd (Beijing, China). Dulbecco’s modified Eagle medium (DMEM) and penicillin-streptomycin (PS) were acquired from Gibco. Fetal bovine serum (FBS) was acquired from Zhejiang Tianhang Biotechnology Co., Ltd. Dilinoleyl-Dil (Cell membrane orange‒red fluorescent probe), DAPI staining solution and YF®488-Phalloidin were all acquired from US Everbright Inc. Streptozotocin (STZ) was acquired from Yuanye (Beijing) Biotechnology Co., Ltd. Ultrapure water was made in the laboratory.

### Isolation and characterization of PA-ELNs

PA-ELNs were isolated by the differential velocity centrifugation approach. In detail, PA powders was soaked in PBS solution (w/v, 1/10) at 4 °C for 4 h. Subsequently, the suspension was centrifuged at 3000×g and 10,000×g for 10 min and 1 h respectively by an Allegra 64R High Speed Refrigerated Centrifuge (Beckman Coulter Life Sciences, California, USA), to remove impurities and cell debris. To obtain PA-ELNs, the supernatant was further centrifuged using an Optima XPN-100 Ultracentrifuge (Beckman Coulter Life Sciences, California, United States) at 100,000×g for 2 h, resuspended in PBS and filtered with a 0.22 μm syringe filter. The PA-ELNs suspension was maintained at -80 °C until subsequent experimental use.

The obtained PA-ELNs were diluted to 1:10 with PBS. Then, the particle size was determined by a NanoSight NS300 Nanoparticle Particle Size Analyzer (Malvern Instruments Limited, Malvern, UK). The hydrodynamic diameter and surface zeta potential of PA-ELNs were measured by a Litesizer 500™ particle size analyzer (Anton Paar, Graz, Austria). To verify the vesicle structure, the morphology of PA-ELNs was observed by a JEM-1400 Plus transmission electron microscope (JEOL Ltd., Akishima, Japan) with phosphotungstic acid staining.

### Proteomic analysis of PA-ELNs

Proteomic analysis by LC-MS/MS was implemented to characterize the protein contained in PA-ELNs. The BCA Protein Assay Kit was used to determine the total protein concentration of PA-ELNs. Lysed PA-ELNs with Tris/SDS/deoxycholate buffer for 15 min were set as samples for subsequent SDS‒PAGE identification. A 12.5% running gel and a 4% stacking gel were first formulated. A 10 µL sample solution diluted with protein loading buffer (5x) and the protein marker were added into the wells. The gel ran for the first 15 min at 80 volts and another 90 min at 110 volts. Finally, the gel was dyed with Coomassie brilliant blue fast staining solution for 30 min, and the result was quickly imaged with a Gel Doc XR+ Imaging System (BIO-RAD, California, USA) [23].

Nano LC-MS/MS technology-based proteomics was further employed to comprehensively identify the protein composition of PA-ELNs. Fifty micrograms of protein extracted from PA-ELNs were firstly reductively alkylated with DL-dithiothreitol and iodoacetamide solutions. After being digested with trypsin, the samples were further desalted and evaporated to dryness. Finally, the samples were redissolved in an aqueous solution containing 0.1% formic acid and 2% acetonitrile in preparation for spectrometry. An Easy-nLC 1200 Nanoflow UPLC and a Q Exactive™ Hybrid Quadrupole-Orbitrap™ Mass Spectrometer (Thermo Fisher Scientific, Massachusetts, USA) were used for analysis. Five microliters of the peptide mixture were used for analysis, and the nanocolumn was a home-made column packed with Acclaim PepMap RPLC C_18_ (1.9 μm, 100 Å, Dr. Maisch GmbH, Germany). The mobile phase consisted of 0.1% formic acid in water (A) and 0.1% formic acid in water and 80% acetonitrile (B). The mobile phase gradient was set as follows: B phase was 4% at 0 min, rose to 8% at 2 min, rose to 28% at 45 min, rose to 40% at 55 min, and rose to the highest concentration of 95% at 56 min and maintained for 10 min. The flow rate was maintained at 600 nL/min. Positive ion mode and data dependent mode were selected. The full MS scan range was from 300 to 1800 m/z, the resolution was at 70,000, the normalized collision energy was at 28.0, and the activation time was at 66 s. The top 20 most intense peptide ions were fragmented by HCD mode. The raw MS files were analyzed and searched against target protein database based on the species of *Periplaneta americana* L. using MaxQuant v1.6.2.10. Specifically, proteins were modificatied to carbamidomethylation (C) (fixed), oxidation (M) (variable) and acetyl (Protein N-term) (variable). The enzyme specificity was set to trypsin and the maximum missed cleavages were set to 3. Meanwhile, the precursor ion mass tolerance and MS/MS tolerance were both set to 20 ppm, respectively.

### Small RNA-Seq analysis of PA-ELNs

Total RNA was isolated using an NGB-58000 exosomal RNA isolation kit (Norgen Biotek, Canada), and the RNA concentration was determined by a NanoDrop spectrophotometer (Thermo Fisher Scientific, Massachusetts, USA). Meanwhile, RNA profiles were profiled using an Agilent 2100 Bioanalyzer (Agilent, California, USA) [24]. Subsequently, small RNA sequencing libraries were created with TruSeq Small RNA Sample Prep Kits (Illumina, San Diego, USA) according to the manufacturer’s protocol. After preparation, the library was single-end sequenced using a Hiseq2000/2500 next generation sequencer (Illumina, San Diego, USA) with a read length of 1x50 bp. The raw data were analyzed with ACGT101-miR v4.2 (LC Sciences, Houston, USA). Clean data were obtained by removing 3’ linkers and junk sequences, and retained sequences with base lengths of 18–26 nt. By comparison with the mRNA (https://www.ncbi.nlm.nih.gov/genome), (http://rfam.janelia.org (http://www.girinst.org/repbase) databases, valid data were obtained and finally aligned with precursors and genomes for miRNA identification. TargetScan v5.0 and miRanda v3.3a were used to target genes of miRNA and the screening criteria were TargetScan score≥50 and miranda energy<-10. The final miRNA target genes were the intersection of the above screening results. To further establish the link between miRNA and function, Gene Ontology (GO, http://www.geneontology.org) and Kyoto Encyclopedia of Genes and Genomes (KEGG, http://www.genome.jp/kegg/) annotations were performed on miRNA target genes [25].

### Cell culture

Mouse fibroblasts (L929), human umbilical vein endothelial cells (HUVECs) and mouse leukemia cells of monocyte macrophages (Raw264.7) were all cultured in DMEM, which contained 10% (v/v) heat-inactivated FBS and 1% (v/v) PS. These cells were maintained in a 37 °C incubator containing 5% CO_2_.

### Effects of PA-ELNs on the proliferation and migration of HUVECs and L929 cells

The effects of PA-ELNs on the proliferation of L929 cells and HUVECs, which are both involved in the wound healing process, were evaluated. Cells at density of 5x10^3^ cells/well were seeded in 96-well plates overnight and treated with PA-ELNs in the protein concentration range of 5 ~ 150 µg/mL for 24 h. Meanwhile, untreated cells served as a control group. Subsequently, the CCK-8 kit was used to detect cells viability according to the instructions.

Additionally, the scratch assay was employed to evaluate the effects of PA-ELNs on L929 migration. L929 cells were seeded onto 12-well plates (1x10^5^ cells/well) and incubated overnight to adhere. Then, scratch wounds were created with 200 µL sterile pipette tips and washed with PBS twice [26]. DMEM containing 100 µg/mL PA-ELNs was added for further culture. Cells of the control group were incubated with the same conditions as above except that PA-ELNs was added. Cell migration was observed and photographed at the 24 and 48 h time points by an inverted microscope under a 10x microscope.

### Cellular internalization of PA-ELNs in L929, HUVECs and Raw264.7 cells

To detect the cellular uptake of PA-ELNs, dilinoleyl Dil with orange‒red fluorescence was employed to label PA-ELNs. Cells were incubated with dilinoleyl Dil-labeled PA-ELNs for 3 h. The treated cells were fixed in 4% paraformaldehyde for 10 min. The nuclei were labeled with DAPI (blue fluorescence), while cytoskeleton were labeled with phalloidin (green fluorescence). Meanwhile, anti-fluorescence quenching seal solution was added to slow fluorescence quenching. Finally, the cells fluorescent images were visualized by a TCS SP8 confocal laser scanning microscopy platform (Leica, Germany) [27, 28].

### Animals

ICR male mice (6 ~ 8 weeks old, 18–20 g) were acquired from SPF Biotechnology Co., Ltd. (Beijing, China). Mice were accommodated in a controlled habitat for 1 week and supplied with food and water ad libitum. Mice were placed in stainless steel cages with 3 mice per cage in a clean room with a light-dark (12:12) cycle. All animal studies were conducted under the guidance of the protocols approved by the animal welfare committee of Chengdu University of Traditional Chinese Medicine.

### Wound healing effects of PA-ELNs on full-thickness diabetic wound model

Male mice were injected intraperitoneally with streptozotocin solution (STZ, 50 mg/kg) for 5 days. After an interval of 3 days, the blood glucose level in the tail vein of the mice was measured with a Roche glucometer. Diabetics was successfully induced in mice when the blood glucose level was ≥ 16.7 mmol/L and persisted for two weeks. Mice without STZ injection served as the DM(-) control group. Both the diabetic mice and normal mice were anesthetized in preparation for cutting a 1 cm diameter circular full-thickness wound on the mouse back. Specifically, 50 µL of PA-ELNs suspended in normal saline with a concentration of 2 mg/mL was uniformly dripped onto the wound site, to ensure all area of wound could be covered by PA-ELNs suspension. Normal mice without STZ treatment were only administrated normal saline. All mice were kept in separate cages. Wound areas were imaged at days 0, 4, 8, 12 and 16 postoperatively, and wound closure rates were calculated by ImageJ 1.46r software [29]. On day 16, the mice were sacrificed. Wound tissues were harvested. The levels of TNF-α and IL-6 in regenerated skin tissue were determined by enzyme-linked immunosorbent assay (ELISA). Briefly, the skin tissue was homogenized and the supernatant was collected. Cytokine levels in the skin tissue homogenates obtained on day 16 after trauma were determined using ELISA kits (Thermo Fisher Scientific, Massachusetts, USA) [17, 30].

### Histological and immunohistochemical analysis

Wound tissues were fixed in 4% paraformaldehyde solution at RT and embedded in paraffin for sectioning [31]. Tissue Sections) were mounted on slides for histological analysis including hematoxylin and eosin (H&E) and masson’s trichrome staining, to visualize epidermal regeneration and collagen formation at day 16.

For immunohistochemical staining, the wound tissue sections were first deparaffinized, rehydrated, and blocked with 3% H_2_O_2_. Subsequently, the sections were incubated with primary antibodies against α-SMA (GB111364, 1:1500, Servicebio), VEGF (GB13034, 1:400, Servicebio), CD31 (GB113151, 1:600, Servicebio), CD68 (gb113109, 1:200, Servicebio), and CD90 (GB11182, 1:1000, Servicebio). After overnight incubation, the sections were treated with HRP goat anti-rabbit (GB23303, 1:200, Servicebio) and developed with DAB (G1211, Servicebio). Nuclei were counterstained with hematoxylin and image acquisition was performed under an E100 microscope (Nikon, Japan) [32].

### Western blotting analysis

The regenerated skin tissue was added to protease inhibitors in advance, and lysed on ice for 30 min with a 10-fold volume of lysis buffer. The samples were added with 5x reduced protein loading buffer and denatured in a boiling water bath for 15 min. Proteins were transferred to PVDF membranes by SDS‒PAGE electrophoresis for further immunoreactions. According to the antibody instructions, the membranes were incubated with P62 (66184-1-Ig, 1:1000, Proteintech Group, Inc.), Beclin-1 (66665-1-Ig, 1:1000, Proteintech Group, Inc.), MMP-9 (GB12132-1, 1:1000, Servicebio) or β-Actin (GB12001, 1:2000, Servicebio). After overnight incubation, they were treated with HRP-goat anti-mouse (GB25301, 1:5000, Servicebio). The optical density value of the target protein band was analyzed by the AlphaEaseFC software processing system [33, 34].

### Statistical analysis

The results are expressed as the mean ± standard deviation (SD). Data analysis was performed in GraphPad Prism 5.0 (GraphPad Software 8, La Jolla, California), and statistical comparisons between groups were performed using one-way analysis of variance (ANOVA). Values of *p* < 0.05 were accepted as statistically significant differences.

## Results and discussion

### Characterization of PA-ELNs

**Fig. 1:**
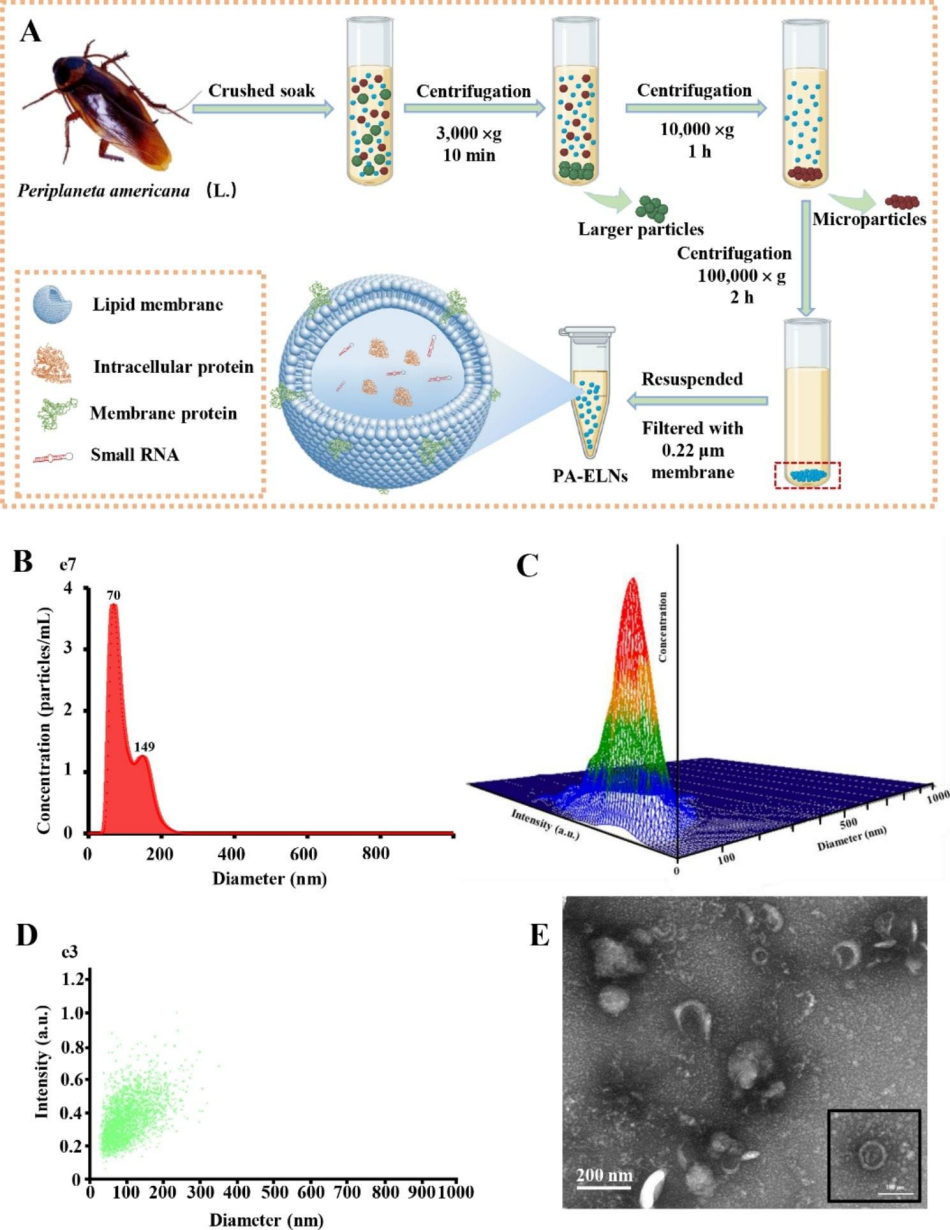
Characterization of exosome-like nanoparticles from *Periplaneta americana* (PA-ELNs). **(A)** Schematic diagram of the extraction and purification of PA-ELNs. The particle distribution **(B)**, 3D plots **(C)** and graphs **(D)** of PA-ELNs by NTA. **(E)** PA-ELNs morphology were visualized by TEM.

The extraction procedure of PA-ELNs is illustrated in Fig. 1A by the differential velocity centrifugation method. Differing from the direct centrifugation of fresh plant or liquid solutions such as milk and juice, the dried PA powder was primarily soaked with precooled PBS buffer. Then, the suspension was successively centrifuged at different centrifugal speeds, until PA-ELNs were obtained and resuspended in PBS buffer.

NTA was further used to analyze particle concentration and particle size. The average particle size of PA-ELNs in Fig. 1B-D is approximately 104.7 nm, the main peak is approximately 70 nm, and the measured particle concentration is approximately 2.33x10^9^±6.35x10^7^ particles/mL. The zeta potential of PA-ELNs measured by DLS was − 9.55 ± 1.77 mV. TEM images showed that the PA-ELNs exhibited a cup-shaped or saucer-shaped structure with a lipid bilayer-bound membrane, and resembled exosome structure of turmeric root or mammalian cells [35](Fig. 1E). As expected, PA-ELNs were found and obtained from the medicinal insect PA for the first time, with similar size and structural characteristics, compared with exosomes from some other natural sources [4, 36–39].

### Protein cargoes of PA-ELNs by proteomics analysis

**Fig. 2:**
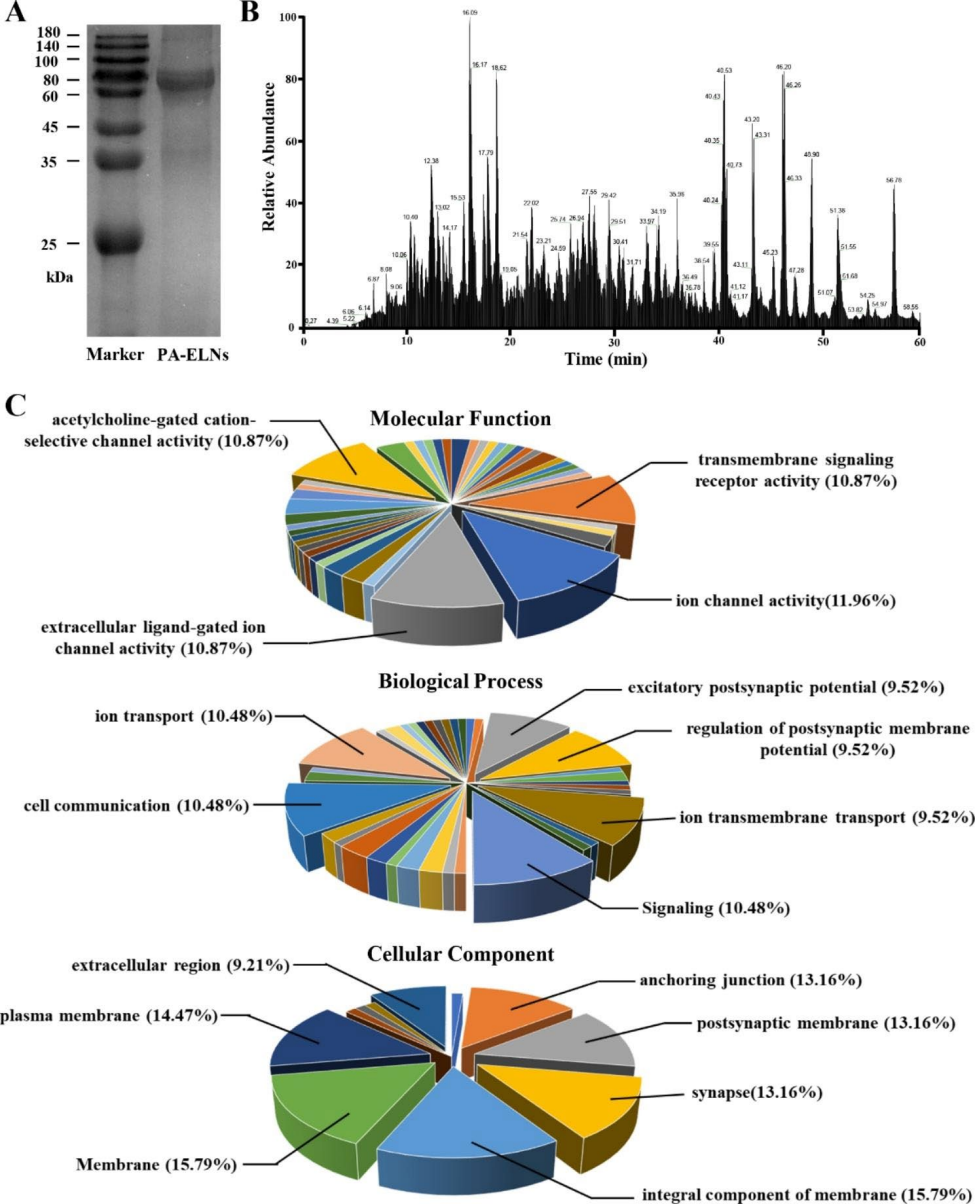
Protein analysis for PA-ELNs. **(A)** The total protein of PA-ELNs was separated by SDS‒PAGE. **(B)** Protein in PA-ELNs was analyzed by proteomics based on LC‒MS/MS technique. **(C)** Gene Ontology analysis of PA-ELNs protein cargoes in molecular function, cellular component and biological process

PA-ELNs exhibited the higher membrane protein in comparison to ELNs from other plant sources. The protein concentration of PA-ELNs measured by the BCA assay was 2.33 ± 0.44 mg/mL. SDS‒PAGE showed that the molecular weight of protein in PA-ELNs was mainly concentrated in the range from 60 to 80 kDa (Fig. 2A). In depth, specific protein varieties were identified from PA-ELNs based on LC‒MS/MS techniques. The total ion chromatogram is shown in Fig. 2B, and a total of 31 proteins were identified (Table S1). Meanwhile, GO analysis was performed to predict the possible roles of these proteins in molecular function, cellular components and biological processes. As reflected in Fig. 2C, in terms of cellular components, proteins were mainly associated with the plasma membrane, membrane composition, postsynaptic membrane, and anchoring junction. The results of biological process analysis showed that the proteins were mainly involved in ion transport including transmembrane transport, cell communication and signal transmission. In addition, molecular functions mainly included ion channel activity, transmembrane signaling receptor activity and acetylcholine-gated cation-selective channel activity. The above analysis results resembled GO analysis of the protein from human neural stem cell-derived extracellular vesicles and T. pisiformis cysticercus‑derived exosome‑like vesicles, especially in terms of cell composition, most of which are related to membrane composition [25, 40].

### Small RNA-seq data analysis of PA-ELNs

**Fig. 3:**
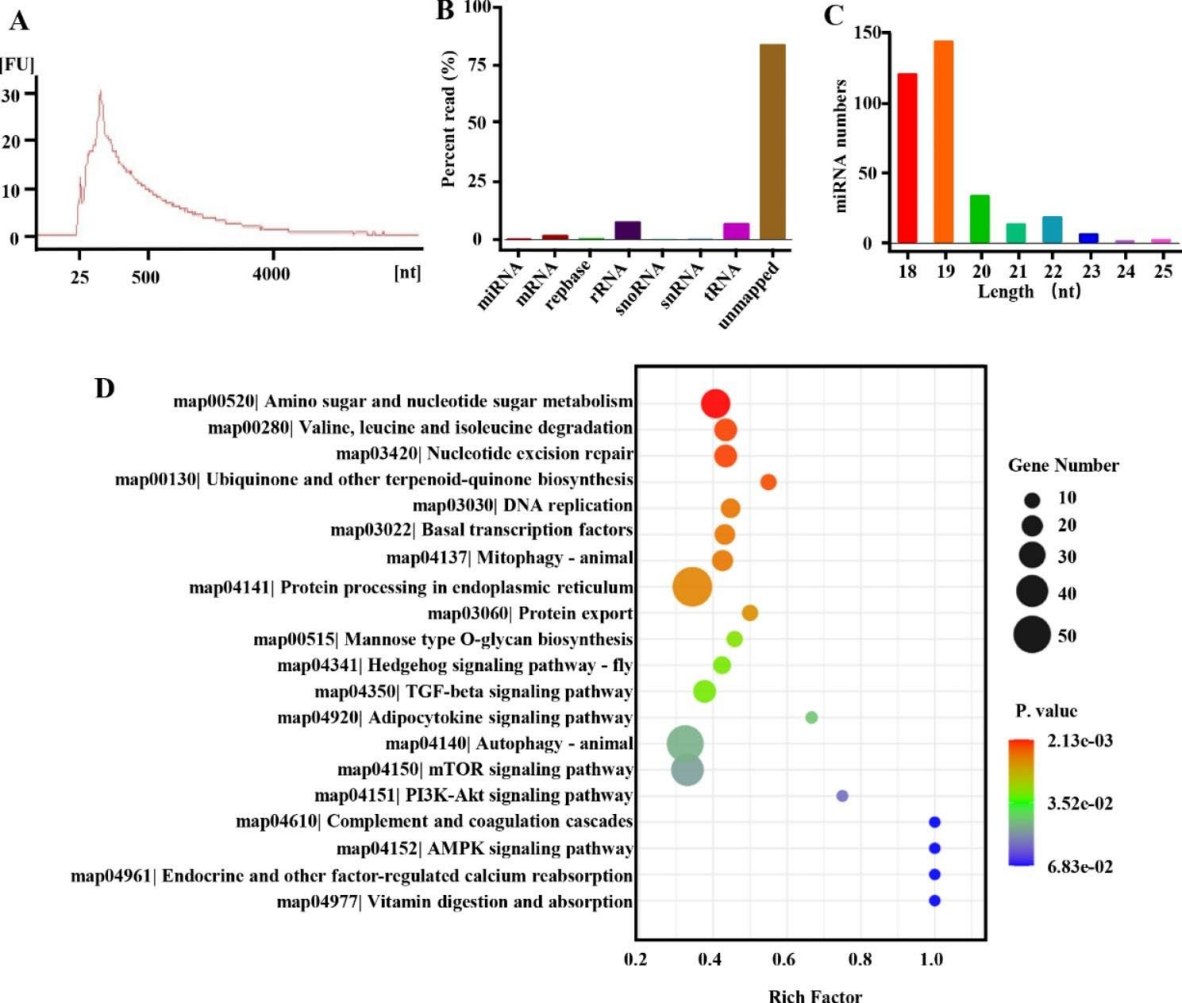
**(A)** Quality analysis results of RNA extracted from PA-ELNs. **(B)** All mapped clean reads are annotated and the percentage is calculated. **(C)** Length distribution of identified miRNAs. **(D)** KEGG enrichment scatter plot of predicted target genes of miRNAs in PA-ELNs.

The total RNA concentration of PA-ELNs was 44.6 ng/mL. The bioanalyzer results are shown in Fig. 3A, the RIN value was 2.2 and the peaks of ribosomal RNAs were not observed, indicating that RNA isolated from PA-ELNs was rich in small RNAs [24]. RNA contained in PA-ELNs was further analyzed by small RNA high-throughput sequencing. A total of 20,760,418 raw reads were identified, and after screening and processing, 6,062,452 (29.20%) clean reads were obtained. RNA categories, including miRNA, tRNA, rRNA, snRNA, and snoRNA, were identified by comparison with mRNA, RFam, and Repbase databases (Fig. 3B). In addition, the RNA categories identified in the Repbase database can be queried separately in the supplement (Fig. S1). miRNA identification was carried out based on miRBase and the *P. americana* genome, as shown in Fig. 3C and Tab.S2. A total of 334 miRNAs were identified, including 54 known miRNAs and 280 novel miRNAs. The length statistics of the identified miRNAs showed that the lengths of miRNAs identified from PA-ELNs were mainly concentrated in 18–19 nt. Meanwhile, the miRNA expression level was calculated by ACGT101-miR, among which there were 45 highly expressed miRNAs (Tab. S3).

### Bioinformatics analysis of miRNAs of PA-ELNs

The potential biological function of highly expressed miRNAs in PA-ELNs was analyzed by GO and KEGG analysis. GO analysis (Fig. S2) predicted the possible roles of these miRNAs in three categories. In terms of biological processes, miRNAs were mainly related to biological processes, metabolism, cellular transcription, signal transduction, material transport, etc. It is worth noting that miRNAs were also associated with vesicle-mediated phagocytosis (GO:0006909) and transport (GO:0016192). In the category of cellular component, miRNAs were mainly involved in membrane composition, cytoplasm, extracellular spaces and extracellular vesicles (GO:0070062). In molecular functions, they were mainly involved in binding to proteins, ATP, metal ions, and nucleic acids.

Meanwhile, functional annotation was performed based on the KEGG database (Fig. S3). The miRNAs isolated from PA-ELNs were involved in different signaling pathways and different metabolic functions. The KEGG enrichment scatter plot (Fig. 3D) showed that these miRNAs were more significantly enriched in the Hedgehog, TGF-beta, autophagy-animal and mTOR signaling pathway. Coincidentally, the research indicated the Hedgehog signaling pathway is related in regulating fibroblast migration during skin wound healing [41]. It is well known that the TGF-beta signaling pathway has pleiotropic effects on wound healing and is one of the most studied signaling pathways in wounds [42, 43]. Moreover, the mTOR signaling pathway has also been reported to be associated with epithelial migration and proliferation [44]. Autophagy is a lysosome-dependent self-renewal mechanism involved in the regulation of various cellular functions. It is closely related to various diseases and plays a role in various processes of wound healing [45]. Thus, the results suggested that these miRNAs contained in PA-ELNs might contribute to the wound healing benefits of PA.

### In vitro pro-proliferative and pro-migratory activities of PA-ELNs

**Fig. 4:**
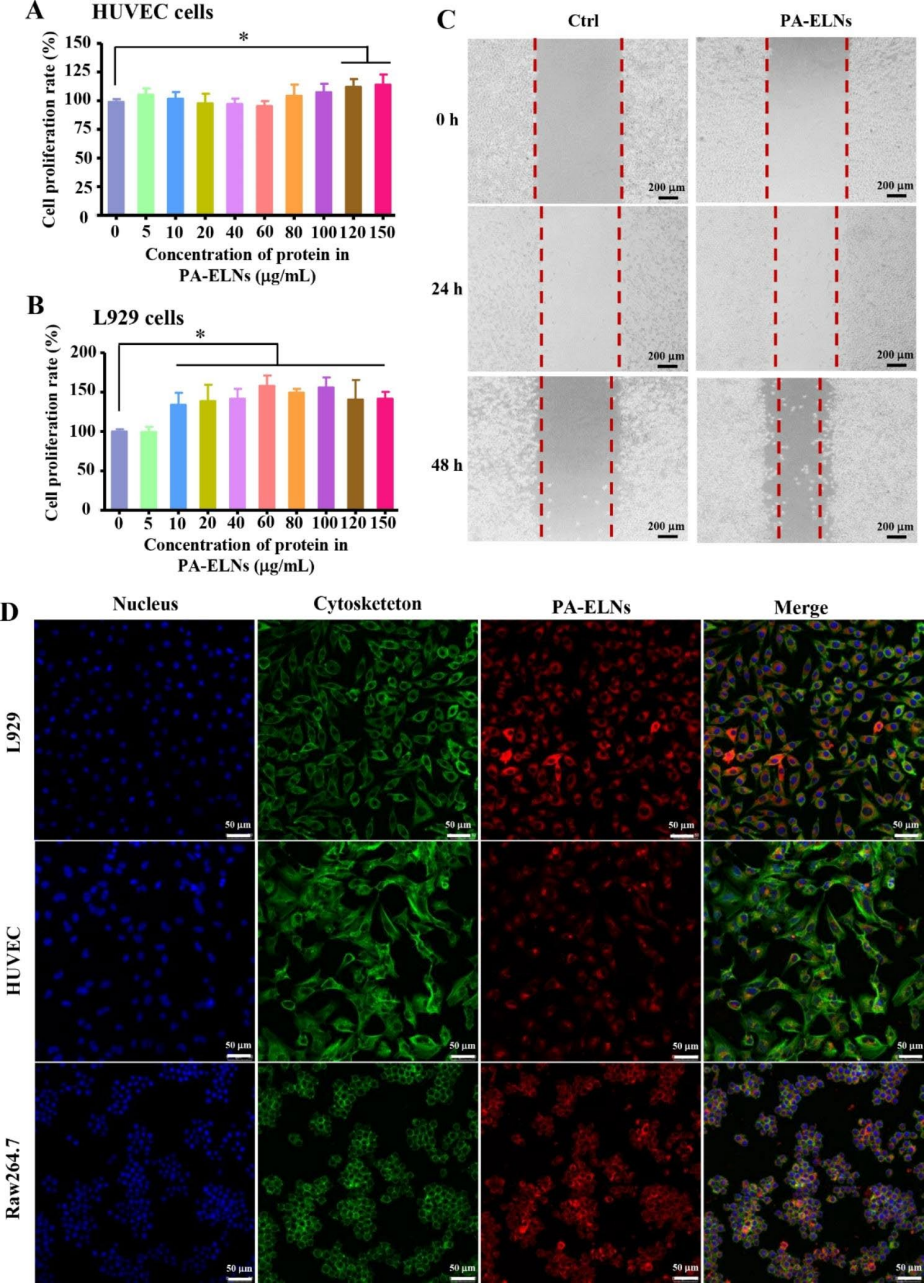
Effects on the proliferation of HUVECs **(A)** and L929 cells **(B)** treated with various concentrations of PA-ELNs for 24 h. ^*^P<0.05 *versus* the control group. **(C)** Wound scratch profiles of L929 cells with or without PA-ELNs treatment for 24 and 48 h. **(D)** Representative confocal microscopy images of cellular uptake profiles of dilinoleyl Dil-labeled PA-ELNs in HUVECs, L929 cells and Raw264.7 cells for 3 h of incubation

During the wound healing process, fibroblasts proliferate through mitosis, and secrete quantities of collagen fibers and matrix components to constitute granulation tissue together with new capillaries to fill the wound tissue defect. Therefore, both fibroblast proliferation and neovascularization act vital roles in wound healing. The proliferation of HUVECs and L929 cells on PA-ELNs for 24 h was investigated by CCK-8 assay. As reflected in Fig. 4A and B, various concentrations of PA-ELNs showed the pro-proliferative capacities of HUVECs and L929 cells. PA-ELNs at concentrations of 120 µg/mL and 150 µg/mL significantly promoted the proliferation of HUVECs (P<0.05), while L929 cells were also significantly promoted at all concentrations except 5 µg/mL (P<0.05). Importantly, when the concentration of PA-ELNs was 60 µg/mL and 100 µg/mL, the average proliferation rate of L929 cells reached 157.90% and 155.88%, respectively. In view of fibroblasts in damaged wounds driving wound contraction and promoting re-epithelialization, the promotion effects on cell migration of PA-ELNs were evaluated by wound scratch assay. PA-ELNs at a concentration of 100 µg/mL were chosen for the migration assay of L929 cells. The results in Fig. 4C showed that compared with the control group, PA-ELNs obviously promoted the migration of L929 cells within 48 h in a time-dependent manner. These results suggested that the topical application of PA-ELNs could contribute to wound healing effects.

### Internalization of PA-ELNs in Raw 264.7, L929 cells and HUVECs

It is believed that when exosomes enter recipient cells via membrane fusion and endocytosis, the internal bioactive cargoes in exosomes can exert biological functions in recipient cells [46]. Apart from fibroblasts and new capillaries, macrophages are also important in the whole process of wound repair, to regulate the inflammatory reaction. Herein, PA-ELNs were labeled with dilinoleyl Dil dye (an orange‒red fluorescence dye) and then their uptake profiles were tracked in three cells lines, Raw264.7 cells, L929 cells and HUVECs. The mean fluorescence intensity values in three cells lines were increased, along with the extended incubation time. As reflected in Fig. 4D, after incubation for 3 h, with the cytoskeleton labeled, Dil-labeled PA-ELNs could be obviously internalized by L929, HUVECs, and Raw264.7 cells. In particular, the red fluorescence derived from the internalized dilinoleyl Dil-labeled PA-ELNs in L929 and Raw264.7 cells was considerably evident, mainly distributed around the nucleus. This result suggested that PA-ELNs were apt to enter these wound healing related cells. These results demonstrated that PA-ELNs could be endocytosed by wound healing-related cells, and exhibit pro-proliferative and pro-migratory activities in vitro.

### Wound healing promotion of PA-ELNs on full-thickness diabetic wound model

**Fig. 5:**
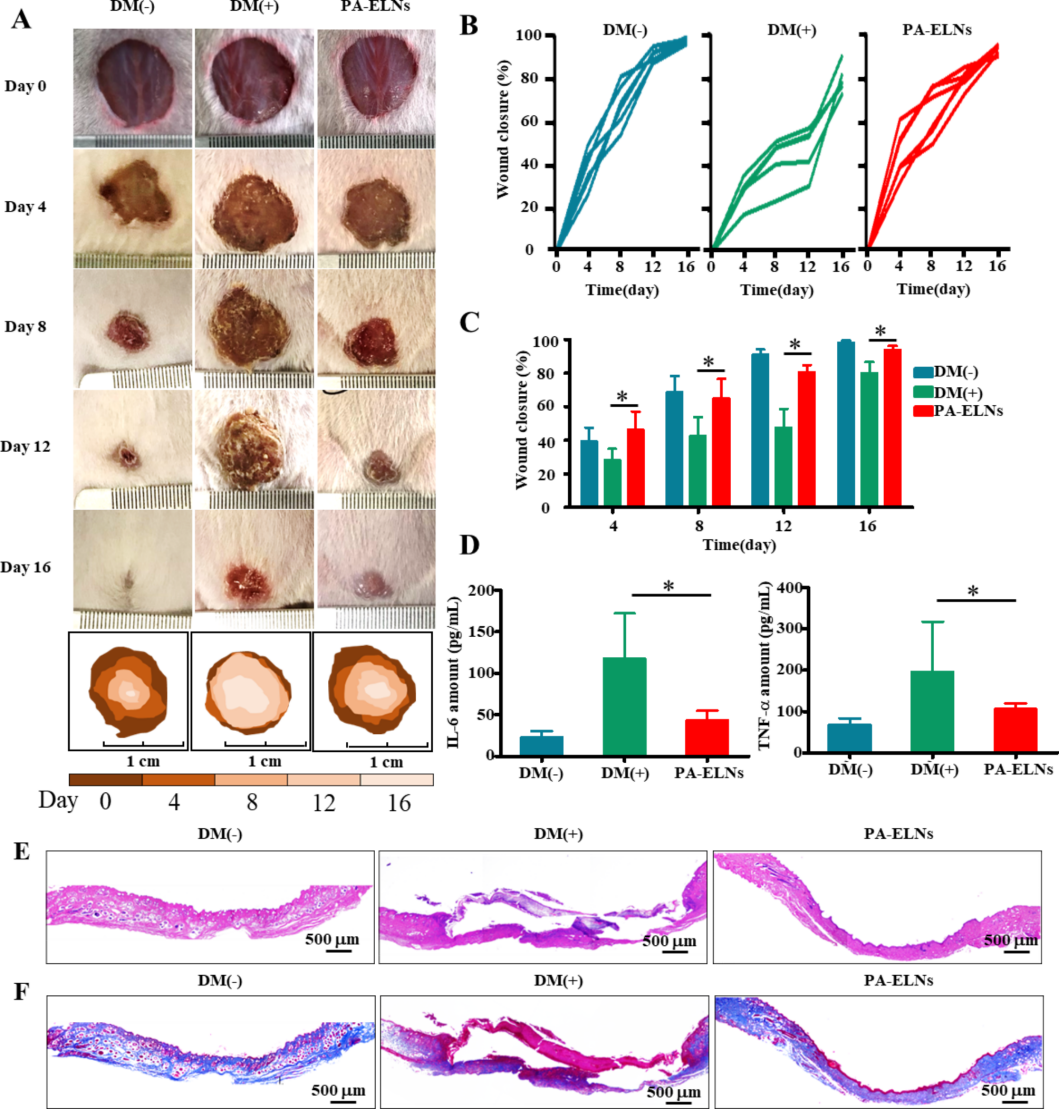
Diabetic mouse full-thickness skin wounds were treated with PA-ELNs. **(A)** Representative pictures of wounds in each group on days 0, 4, 8, 12 and 16. Wound closure rates of each group over 16 days **(B, C)**. **(D)** Levels of IL-6 and TNF-α in wound biopsy at Day 16 by ELISA determination. ^*^P<0.05 *versus* the DM(+) group. Representative images of wound biopsy at Day 16 by H&E staining **(E)** and Masson’s trichrome staining **(F)**

In order to further evaluate the wound healing promotion effect of PA-ELNs in vivo, a diabetic mouse full-thickness wound model was established. Meanwhile, to reveal the chronic healing characteristics of wounds manifested by diabetics, STZ-induced diabetic mice and the uninduced mice were employed as the positive model group (DM(+)) and negative control group (DM(-)), respectively. As reflected in Fig. 5A, the wounds in each group had a certain degree of shrinkage after 16 days. The wounds in the DM(-) group showed almost complete healing, whereas the wound healing of the DM(+) group was significantly delayed. With the administration of PA-ELNs, the wound area was significantly smaller than that in the DM(+) group at the same time-point. PA-ELNs could effectively promote wound healing and induce significant wound-healing effect. The wound closure rate (Fig. 5B C) also showed that mice treated with PA-ELNs suffered higher wound closure rate, than mice in the DM(+) group on days 4, 8, 12 and 16 (P<0.05). The average wound closure rate of mice in the PA-ELNs group reached 96.78% on day 16, which was close to complete healing. Furthermore, beyond visual wound healing, expression of pro-inflammatory factor (IL-6 and TNF-α) in wound biopsies at day 16 was measured by ELISA. The results showed (Fig. 5D) that the overexpression of IL-6 and TNF-α in the regenerated skin tissue of DM(+) mice could be effectively decreased by treatment with PA-ELNs on the 16th day.

### PA-ELNs promoted epithelial regeneration and collagen production

Hematoxylin and eosin (H&E) and Masson’s trichrome staining were served as the wound-healing progress assessment after PA-ELNs treatment. The H&E staining results (Fig. 5E) showed that on day 16, the re-epithelialization of the new skin tissue in the DM (-) group and PA-ELNs group had been basically completed. However, the epidermal layer in mice of the DM (+) group was still not generated completly. The amounts of fibroblasts were tightly packed in the PA-ELNs group, indicating an ideal wound healing status. Collagen is the structural component of the extracellular matrix, playing an important role in wound repair. After Masson’s trichrome staining, collagen deposition in skin tissues was observed. As shown in Fig. 5F, compared with the DM (+) group without treatment, PA-ELNs promoted the regeneration of collagen (blue area) in wounds, which was similar to the abundant collagen deposition in the DM (-) group. The DM (+) group showed a loose and sparse collagen distribution, however, collagen deposition in the PA-ELNs group displayed a more orderly distribution. These results illustrated that the wound healing effects of PA-ELNs are involved in relieving inflammation, and promoting re-epithelialization and collagen production.

### PA-ELNs regulated angiogenesis and cytokine secretion in wound healing

**Fig. 6:**
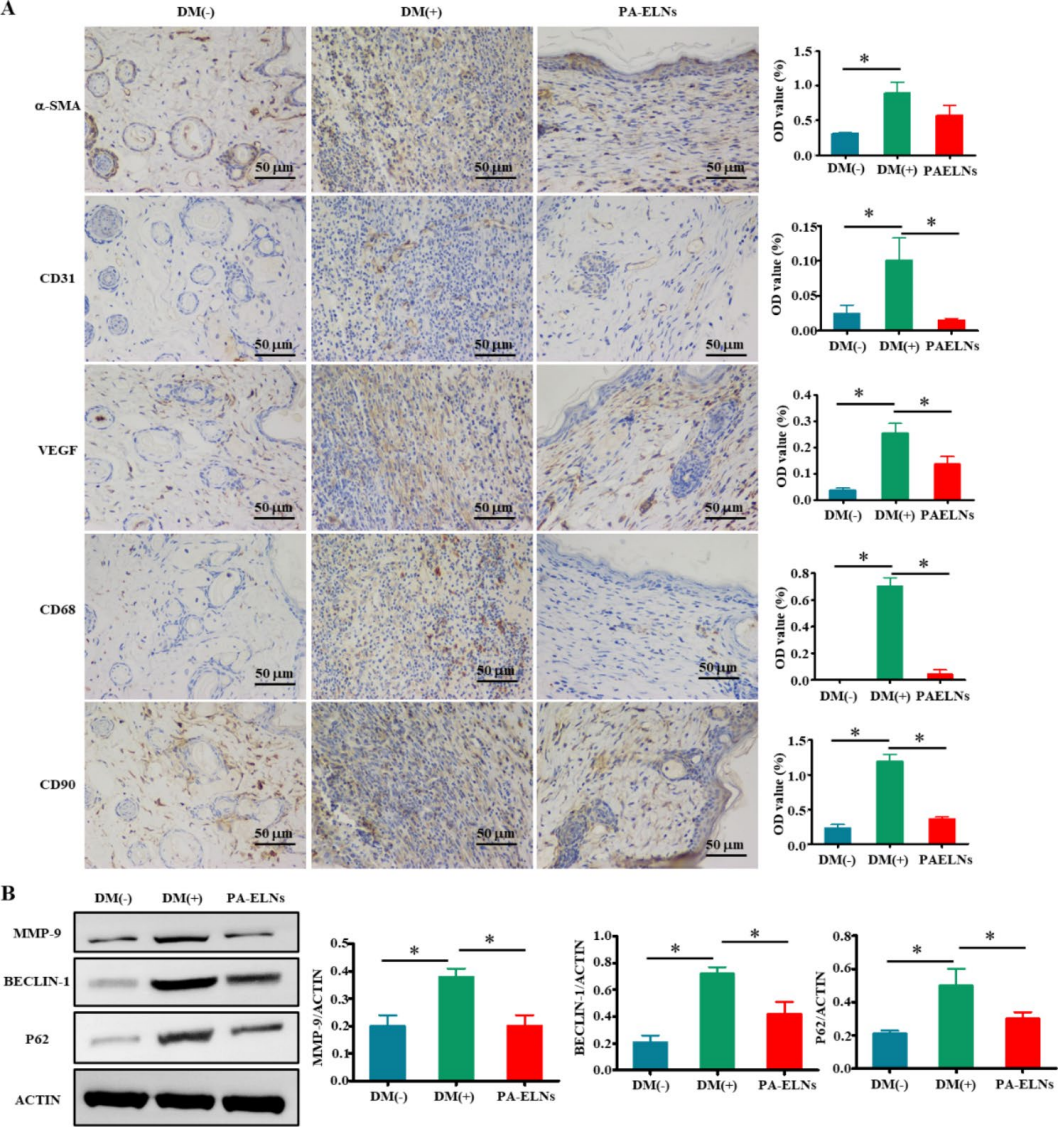
**(A)** The expression of α-SMA, CD31, VEGF, CD68 and CD90 in the regenerated mouse skin tissue in each group on the 16th day was determined by immunohistochemical staining. **(B)** Western blot results of MMP-9, BECLIN-1 and p62 protein expression in wound tissue of mice at day 16. ^*^P<0.05 *versus* DM(+) group

As shown in Fig. 6A, compared with the DM(-) group, CD68 had a high level of expression in the DM(+) group on the 16th day, which was suppressed after treatment with PA-ELNs, indicating that PA-ELNs blocked persistent inflammatory responses. In this study, PA-ELNs reduced the over-expression of CD90 and α-SMA on the 16th day, which was beneficial for preventing excessive fibrosis. In addtion, a major marker in the proliferative stage of wound healing is the generation of vascular network. Pro-angiogenic factors, especially VEGF, bind to endothelial cell receptors to guide blood vessels from the periphery of the wound into the wound bed and stimulate capillaries to form new immature rings and branches. After angiogenesis reaching a fixed point, pro-angiogenic factors become anti-angiogenic growth factors, some angiogenic cells gradually disappear from apoptosis, and the other part becomes a mature vascular network [47]. Meanwhile, CD31 acts a vital role in vascular regeneration and angiogenesis during wound healing [48]. Therefore, the expression of VEGF and CD31 is a dynamic process. It has been reported that in the inchoate stage of wound healing, the expression levels of CD31 and VEGF after drug treatment were significantly upper compared with the control group, while showed lower expression in the later stage [49–51]. In our current study, the wounds of DM (+) mice were basically healed, and the secretion of CD31 and VEGF were both at low levels on the 16th day, with the same trend after treatment with PA-ELNs, while the opposite was true in the DM(+) group. In conclusion, PA-ELNs regulated angiogenesis and cytokine secretion in the late stage, which is beneficial for accelerating wound healing.

### PA-ELNs alleviated inflammation and autophagy dysfunction in wound healing

The handling of redundant or defective organelles in wound healing is very important, and autophagy represents this biological process. Some studies have proved that the progress of wound repair is significantly delayed after treatment with the autophagy agonist rapamycin [52]. Western blot was devoted to measure the secretion levels of the autophagy markers Beclin-1 and p62 in the regenerated skin tissue of mice on the 16th day. As reflected in Fig. 6B, the expression levels of Beclin-1 and p62 were remarkably decreased with PA-ELNs treatment compared with the DM(+) group. These results indicated that PA-ELNS reversed autophagy dysfunction in diabetic mice. In addition, MMP-9 belongs to the zinc-dependent endopeptidase family. In diabetic chronic wounds, excessive expression of MMP-9 leads to ECM decomposition, and then affects cell migration and wound epithelialization. Inhibiting the expression of MMP-9 is beneficial to diabetic wound healing [53, 54]. Similarly, the level of MMP-9 in the regenerated skin tissues of each group was measured by WB on the 16th day. These results showed that PA-ELNs could downregulate the expression of autophagy-related proteins and the expression of MMP-9.

## Discussion

Animal-derived traditional Chinese medicines (TCMs) are central components of TCM. There are some representative animal-derived TCMs, such as bezoar (Bovis calculus), centipede (Scolopendra), musk (Moschus), and donkey-hide glue (Asini Corii Colla), playing an irreplaceable role in clinics for the treatment of tumor, cardiovascular and cerebrovascular diseases, arthritis and so on. According to statistics, the current Chinese Pharmacopoeia (2020 edition) has documented 49 varieties of animal-derived TCMs and even 497 prescription preparations containing animal-derived TCMs [55]. Unfortunately, the complex macromolecular constitutes in animal-derived TCMs have not been thoroughly researched, compared to these bioactive small molecular components in plant-derived TCMs [56]. The previous studies were apt to explore the bioactivities of proteins or peptides in medicinal animal products, such as Centipede, Snake venom, Wasp venoms, Pilose antler [57–59], and Chinese giant salamander. However, in view of these proteins or peptides which are susceptible to degradation and denaturation, or difficult to isolate and characterize their structures, the bioactive material basis attributing to the wound healing benefit of *Periplaneta americana* L. (PA) still needs to explore further.

Exosomes are initially considered as “garbage cans”, and their role in cellular communication and signal transmission is gradually recognized. Particularly, studies have reported that their abilities to participate in the interkingdom communication are highly consistent with the pharmacological action of their source. For example, ginger-derived exosome-like nanoparticles were able to be absorbed by gut bacteria and contained miRNAs that regulated gut bacteria composition to alleviate colitis in mice [60]. Exosomal miRNA from infarcted myocardium can mobilize the transport of bone marrow progenitor cells to achieve myocardial injury repair [61]. Recently, some genetic codes about growth factor in PA have been found. Li et al [22] revealed its developmental mechanism of regeneration ability of amputated limb, by analyzing the genomic and functional landscapes of developmental plasticity. Several RNA information on key roles of decapentaplegic (Dpp) regulation have been found in PA. In view of the role in cell-to-cell communication through RNA transfer or gene/protein expression regulation, we speculated that these RNA in PA-ELNs would activate these growth factor signals to stimulate tissue repair. Therefore, unlike these previous studies to investigate the potential bioactive small-molecules or macromolecules in medicinal animals, we focused on the isolated exosome-like nanoparticles from PA, in which was distinct-different from investigating nucleosides, amino acids or peptides in PA.

Based on these above-results, we have clearly demonstrated that PA-ELNs possessed the significant wound healing effect against DM wounds. In the diabetic wound environment, some important cells such as endothelial cells, fibroblasts, macrophages, and keratinocytes participated in angiogenesis, collagen synthesis, and anti-inflammatory processes, play the important roles in wound healing. Accumulating evidences indicated that these wound accelerators including either epidermal growth factors, natural compounds or macromolecular wound dress would regulate the expression of biologically functional molecules and modulate the afore-mentioned cells through various pathways, so that to promote angiogenesis, collagen synthesis and inhibit inflammation [62]. In this study, PA-ELNs accelerated the transition of the inflammatory phase by inhibiting the expression of pro-inflammatory factors and promoted collagen deposition and epithelialization in the wound site. Furthermore, PA-ELNs also participated in the regulation of autophagy by down-regulating the expression of BECLIN-1 and p62 protein. It is also worthy of note that these wound healing mechanisms of PA-ELNs coincide with the GO analysis of proteins in PA-ELNs and KEGG analysis of miRNAs in PA-ELNs. In addition, exosomes from body sources have been verified the wound healing benefits, by promoting neovascular growth, stimulating collagen deposition, and inhibiting inflammation, in which these exosomal proteins, miRNAs, long noncoding RNA (lncRNA), and circular RNA(circRNA) also have been regarded as the wound healing codes [63]. Even so, how to topically apply these exosomes on wounds still deserves further exploration. Commonly, exosomes were embedded with various hydrogels or dressings as scaffolds to accumulate exosomes on diabetic wounds to synergistically promote wound healing [32, 64–67]. Therefore, to employ PA-ELNs in DM wound clinically, much more researches are required to determine the production routes of PA-ELNs for a large-scale production and design the appropriate scaffolds for local application.

## Conclusions

In this study, exosome-like nanoparticles were successfully isolated from *Periplaneta americana* (L.) (PA-ELNs). Omics and bioinformatics analyses were employed to characterize PA-ELNs and prompted that the potential wound-healing mechanisms related to these miRNA and protein cargos. Furthermore, the local application of PA-ELNs showed the significant advantages to promote diabetic mouse wound healing, which involved in wound inflammation inhibition, and collagen deposition and epithelial re-epithelialization promotion. In summary, in view of the intrinsic amputation regeneration feature of PA and its wound healing benefit, PA-ELNs would exhibit the potential wound therapeutic benefits by means of transmitting these intrinsic macromolecular cargoes.
